# Identification and Quantification of Glucosinolates in Kimchi by Liquid Chromatography-Electrospray Tandem Mass Spectrometry

**DOI:** 10.1155/2017/6753481

**Published:** 2017-02-19

**Authors:** Ho Jin Kim, Mi Jin Lee, Min Hee Jeong, Jang Eok Kim

**Affiliations:** ^1^National Agricultural Products Quality Management Service, Kimchun 740-871, Republic of Korea; ^2^School of Applied Biosciences, College of Agriculture and Life Sciences, Kyungpook National University, Daegu 702-701, Republic of Korea

## Abstract

A novel and simple method for detecting five glucosinolates (glucoalyssin, gluconapin, glucobrassicanapin, glucobrassicin, and 4-methoxyglucobrassicin) in kimchi was developed using liquid chromatography-electrospray tandem mass spectrometry (LC-MS/MS). The chromatographic peaks of the five glucosinolates were successfully identified by comparing their retention times, mass spectra. The mobile phase was composed of A (acetonitrile) and B (water). As for glucosinolate, the relative quantities were found through sinigrin, and five different compounds that have not been previously discovered in kimchi were observed. Monitoring was carried out on the glucosinolate in 20 kimchis distributed in markets, and this study examined the various quality and quantity compositions of the five components. The glucoalyssin content ranged from 0.00 to 7.07 *μ*mol/g of day weight (DW), with an average content of 0.86 *μ*mol/g of DW, whereas the gluconapin content ranged from 0.00 to 5.85 *μ*mol/g of DW, with an average of 1.17 *μ*mol/g of DW. The content of glucobrassicanapin varied between 0.00 and 11.87 *μ*mol/g of DW (average = 3.03 *μ*mol/g of DW), whereas that of glucobrassicin varied between 0.00 and 0.42 *μ*mol/g of DW (average = 0.06 *μ*mol/g of DW). The 4-methoxyglucobrassicin content ranged from 0.12 to 9.36 *μ*mol/g of DW (average = 3.52 *μ*mol/g of DW). A comparison of the contents revealed that, in most cases, the content of 4-methoxyglucobrassicin was the highest.

## 1. Introduction

Glucosinolates (GSLs) are well-known secondary metabolites and are rich in Brassicaceae plants, such as broccoli, all types of cabbages, cauliflower, and Brussels sprouts. More than 132 types of GSLs have been uncovered so far [1]. They share a chemical structure composed of *β*-thioglycoside* N*-hydroxysulfates (also known as (*Z*)-*N*-hydroximinosulfate esters or* S*-glucopyranosyl thiohydroximates), with a side chain R and a sulfur linked *β*-D-glucopyranose moiety, and can be divided into aliphatic, aryl, and indole types depending on the primary amino acids. If the cellular structures of Brassicaceae plants are decomposed, GLS is hydrolyzed by inherent myrosinase (EC3.2.1.147), which decomposes the glucose moiety in the main skeleton. The resulting products can give glucose, and unstable aglycone and aglycone can be rearranged into isothiocyanates (ITCs), nitriles, and other products. Breakdown products are different depending on the reaction conditions and each GLS structure, but GLSs with an aliphatic or aromatic side chain at a neutral pH mainly generate ITCs [2]. Most biological activities of GLSs come from the hydrolysate [3]. Over the past few years, specific degradation products of GLSs have been shown to induce enzyme activity, such as Phase II detoxification enzymes, including quinone reductase, glutathione-*S*-transferase, and glucuronosyltransferases, and be strong cancer prevention agents in various animal experiments [4–6]. Sulforaphane and other ITCs are also estimated to inhibit the cell cycle, promote apoptosis, and prevent tumor growth [7, 8]. They seem to be effective for colorectal cancer [9], lung cancer [10], and possibly prostate cancers [11] in humans.

Many modern analytical methods, such as HPLC, NMR, mass spectroscopy, near-infrared spectroscopy, biosensing [12, 13], and ELISA [14], have been developed. Chromatography is the most widely applied method for the analysis of GLSs. In particular, HPLC with ultraviolet or diode array detection (LC-DAD) [15–19] and LC coupled with mass spectrometry (MS) [20–30] have many applications.

The main ingredient of kimchi is Chinese cabbage, and a GLS content of approximately 8.3 umol/g dry weight is known to be included in Chinese cabbage [31]. In addition, kimchi is a fermented food that is eaten every day by most Koreans and is increasingly consumed around the world, with many studies reporting its efficacy as an anticarcinogenic [32], antioxidative [33, 34], and immune stimulatory activity [34]. However, the GSL content and profile of kimchi, with each GSL from kimchi generating a specific breakdown product that possesses different biological properties, have not yet been studied.

Therefore, this paper analyzed and validated the GLS detected in kimchi and compared it with that of Chinese cabbage to establish an analytical method that is helpful for future research on the biological properties of kimchi. The establishment of this analytical method will help in the study and development of active ingredients of kimchi and will also affect cooked food research on Brassicaceae plants.

## 2. Materials and Methods

### 2.1. Standards and Reagents

Sinigrin and DEAE Sephadex A-25 were purchased from Sigma-Aldrich (St. Louis, MO, USA). Sodium acetate (NaC_2_H_3_O_2_·3H_2_O) was obtained from Hayashi Pure Chemical Industries, Ltd. (Osaka, Japan). HPLC-grade acetonitrile, methanol, and ethanol were purchased from Merck (Darmstadt, Germany). Water was purified using a Milli-Q Rios/Elix water purification system (Millipore, Bedford, MA, USA).

### 2.2. Extraction of Crude Glucosinolates (GSLs) and Their Desulfation

Kimchi samples purchased from local supermarkets were frozen at −40°C and lyophilized for 48 h. Approximately 100 mg of freeze-dried samples was weighed in 2 mL polypropylene-capped microcentrifuge tubes. After 1.5 mL of 70% aqueous methanol was added, an extract was obtained by sonication for 5 min at 70°C. After cooling, the extract was centrifuged at 12,000 ×g for 10 min. The supernatants were removed using a syringe and filtered through a 0.2 *μ*m nylon filter (Waters Associates, Milford, MA). The extraction procedure was repeated two times, and the supernatants were combined. For enzymatic desulfatation of the GSLs, the extract solution was loaded onto a DEAE Sephadex A-25 column and the GSLs were treated in the column with aryl sulfatase (H-1 type from Sigma, St. Louis, MO, USA) following the method of Official Procedure ISO 9167-1 (1992) [35]. Briefly, desulfation of the crude GLS extracts was performed using a DEAE anion exchange column, which was prepared by adding a slurry of DEAE Sephadex A-25 that was previously activated with 0.5 M sodium acetate. Five milliliters of a sinigrin solution (0.1 mg/mL), used as an external standard, was separately desulfated using the same DEAE anion exchange column. The crude GLS extracts were loaded onto a preequilibrated DEAE anion exchange column. After washing with 1 mL (×3 times) of ultrapure water to remove cation and neutral ions, 75 *μ*L of aryl sulfatase (E.C.3.1.6.1) was loaded onto each column. Following a desulfation reaction overnight (16–18 hours) at room temperature, desulfated GLSs were eluted with 0.5 mL (×3 times) of distilled water. The eluate was freeze-dried and stored at −80°C. Prior to HPLC analysis, the residue was dissolved in water and filtered with a 0.45 *μ*m membrane.

### 2.3. HPLC Analysis

HPLC analysis was performed on an Agilent 1200 HPLC system coupled with a photodiode array (PDA) detector (Agilent Technologies, Waldbronn, Germany). The chromatographic column used was a Inertsil ODS-3 column (150 mm × 3.0 mm, i.d. with 3 *μ*m particle diameter, GL Science, Tokyo, Japan) at 40°C. A mobile phase composed of A (acetonitrile) and B (water) with a gradient elution of 0 min (0% A), 0–2 min (0% A), 2–7 min (0–10% A), 7–16 min (10–31% A), 16–19 min (31-31% A), 19–21 min (31–0% A), and 21–27 min (0–0% A) was used in this study. The sample injection volume was 5 *μ*L, and the flow-rate was set at 0.4 mL/min. Peaks were detected at 227 nm.

To quantify the amount of GLS, we used the standard methods reported by ISO 9167-1 (1992). Briefly, individual GSLs were identified in comparison with the retention time of a sinigrin standard. Quantification of individual GSLs was accomplished using the response factors shown in Table 1. Measurements were performed in triplicate.

### 2.4. LC-MS/MS Analysis

The MS data were acquired by electrospray ionization (ESI) mass spectrometry with an API 4000 Q TRAP system (Applied Biosystems, Foster City, CA, USA) in positive ion mode ([M+H]+) that was equipped with an Agilent 1200 series HPLC system. The MS operating conditions were as follows: ion spray voltage (5.5 kV), curtain gas (20 psi), nebulizing gas (50 psi), heating gas (50 psi), high purity nitrogen (N_2_), heating gas temperature (550°C), declustering potential (100 V), entrance potential (10 V), and spectra scanning range (*m/z* 100–1000) (scan time 4.8 sec).

## 3. Results and Discussion

### 3.1. Identification of Glucosinolates

**Figure 1:**
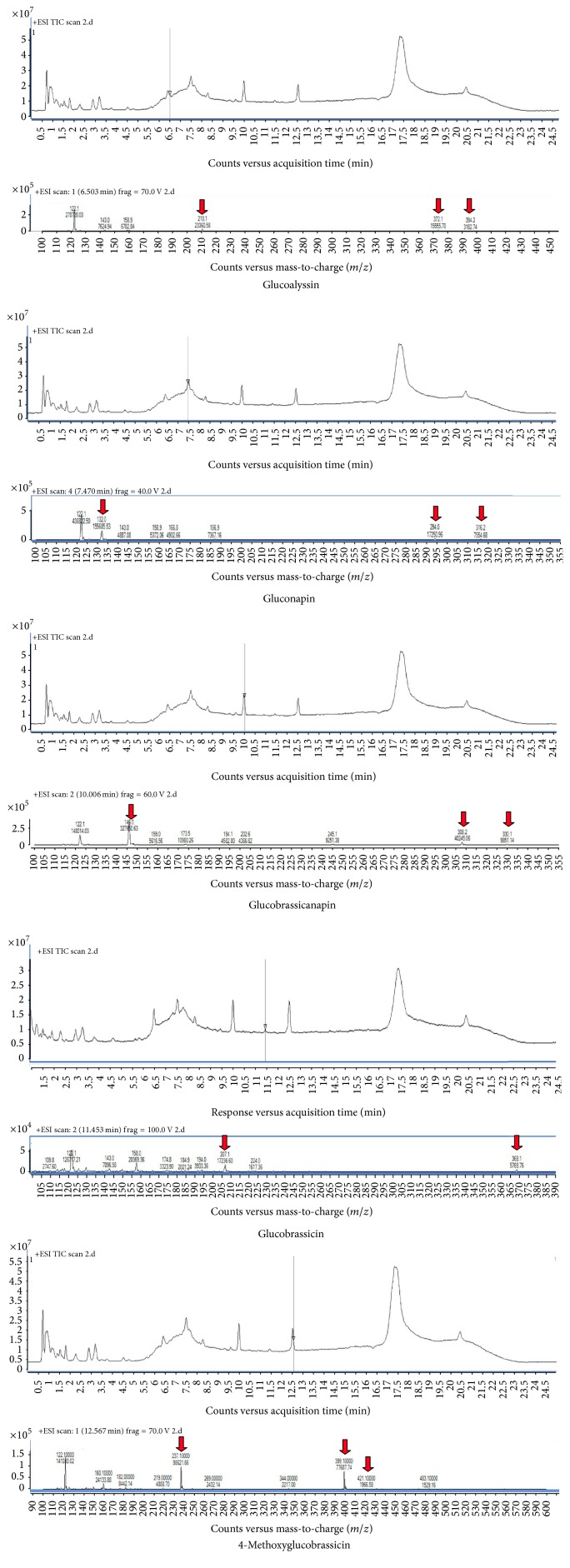
Mass spectrometry of the five glucosinolates (GSLs).

**Figure 2:**
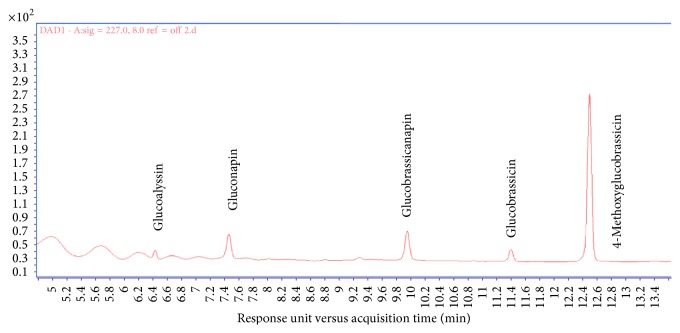
HPLC chromatograms of a mixture of the 5 glucosinolate (GSL) compounds detected at 227 nm: (1) glucoalyssin, (2), gluconapin, (3) glucobrassicanapin, (4) glucobrassicin, and (5) 4-methoxyglucobrassicin.

To understand how kimchi would meet the conditions for GSL analysis, this study carried out an experiment using ISO method 9167-1 (1992). In the experiment, because there are no standardized GSL goods, substances had to be determined according to their retention time, mass spectrum (Table 2, Figures 1 and 2). To begin, the first substance, which was compound 1, yielded a retention time of 6.42, and mass spectrometry yielded a M_DS_ of 371 (a molecule with SO_3_ separated). M_DS_+H-162, M_DS_+H, and M_DS_+Na turned out to be 210, 372, and 394, respectively, which indicated glucoalyssin. In terms of compound 2, the retention time appeared to be 7.48, and according to results of mass spectrometry, gluconapin was confirmed. M_DS_ was 293 (M_DS_+H-162 = 132, M_DS_++H = 294, M_DS_+Na = 316). Regarding compound 3, it had a retention time of 9.84, and in terms of the mass spectrum, the study found M_DS_ of 307, which confirmed the identity of glucobrassicanapin. The retention times of compound 4 and compound 5 were found to be 11.39 and 12.51, respectively, and through mass spectrometry, M_DS_s were found to be 368 and 398, respectively. This was one of the ways that we confirmed the identities of glucobrassicin and 4-methoxyglucobrassicin. In the case of glucobrassicin, two peaks were confirmed because the sensitivity of the peak was low. These peaks were confirmed to be the glucobrassicin because of the main peak.

All compounds were identified by comparing numbers with those in reference [36].

### 3.2. Applications of the Optimized Method

Among the 20 different kimchis that were analyzed in this study, GSLs were detected in kimchi purchased from supermarkets (Table 3). Each sample was analyzed in triplicate. Identification of the five compounds was performed by comparing their retention times, mass spectra (Section 3.1). The qualitative and quantitative compositions of the five compounds in kimchi varied significantly. More specifically, the glucoalyssin content ranged from 0.00 to 7.07 *μ*mol/g of DW, with an average content of 0.86 *μ*mol/g of DW, whereas the gluconapin content ranged from 0.00 to 5.85 *μ*mol/g of DW, with an average of 1.17 *μ*mol/g of DW. The content of glucobrassicanapin varied between 0.00 and 11.87 *μ*mol/g of DW (average = 3.03 *μ*mol/g of DW), whereas that of glucobrassicin was between 0.00 and 0.42 *μ*mol/g of DW (average = 0.06 *μ*mol/g of DW). Finally, the 4-methoxyglucobrassicin content ranged from 0.12 to 9.36 *μ*mol/g of DW (average = 3.52 *μ*mol/g of DW). The contents were compared, and 4-methoxyglucobrassicin tended to be the highest in content. It is worth noting that, in samples A, B, C, I, and S, the content of glucobrassicanapin was observed to be greater than that of 4-methoxyglucobrassicin, and the study noted other substances that had not been previously extracted.

As stated above, the relative quantities of the five different GSLs in kimchi were found, and the sampling method, qualitative method, and quantitative method for simultaneous analytical detection were successfully conducted via RP-HPLC-MS. In addition, this was the first time GSL from kimchi was examined, and eventually, such an achievement will be useful not only for understanding the remarkable effects of kimchi but also for determining organic kimchis from others. The study offered a chance to discover the GSL included in kimchi not only in the forms of the five components but also in the forms of GSL metabolites (thiocyanate, isothiocyanate, and nitrile). The metabolites are known to have even more impressive effects than those of GSL and remain an intriguing research topic.

## Figures and Tables

**Table 1:** Factor value of each of the compounds (ISO 9167-1).

Compound	Factors
Glucoalyssin	1.07
Gluconapin	1.11
Glucobrassicanapin	1.154
Glucobrassicin	0.29
4-Methoxyglucobrassicin	0.25

**Table 2:** Glucosinolates (GSLs) identified by LC-ESI/MS.

Glucosinolate	Molecular formula	M	M-80=M_DS_	Positive ionization		
Trivial name				M_DS_+H-162	M_DS_+H	M_DS_+Na
Glucoalyssin	C_13_H_25_NO_10_S_3_	451	371	210	372	394
Gluconapin	C_11_H_19_NO_9_S_2_	373	293	132	294	316
Glucobrassicanapin	C_12_H_21_NO_9_S_2_	387	307	146	308	330
Glucobrassicin	C_16_H_20_N_2_O_9_S_2_	448	368	207	369	—
4-Methoxyglucobrassicin	C_17_H_22_N_2_O_10_S_2_	478	398	237	399	421

**Table 3:** Glucosinolate (GSL) content (*μ*mol/g DW) in 20 kimchi samples (*n* = 3).

Compound	A	B	C	D	E	F	G	H	I	J
Glucoalyssin	1.42 ± 0.02	7.07 ± 0.07	0.45 ± 1.1	0.29 ± 0.01	1.31 ± 0.02	0.83 ± 0.01	1.29 ± 0.02	0.00	1.67 ± 0.01	0.07 ± 0.01
Gluconapin	4.97 ± 0.03	5.85 ± 0.02	3.05 ± 0.02	0.18 ± 0.00	1.81 ± 0.02	1.11 ± 0.03	1.91 ± 0.01	0.00	1.10 ± 0.02	0.00
Glucobrassicanapin	11.03 ± 0.08	11.87 ± 0.10	7.95 ± 0.06	0.27 ± 0.00	3.38 ± 0.04	2.79 ± 0.04	4.37 ± 0.03	0.00	2.72 ± 0.01	0.00
Glucobrassicin	0.04 ± 0.00	0.42 ± 0.02	0.02 ± 0.00	0.00	0.00	0.04 ± 0.00	0.20 ± 0.01	0.00	0.03 ± 0.00	0.00
4-Methoxyglucobrassicin	6.98 ± 0.02	9.36 ± 0.05	1.62 ± 0.02	1.83 ± 0.04	5.94 ± 0.03	3.18 ± 0.03	6.22 ± 0.05	0.12 ± 0.01	0.33 ± 0.01	1.89 ± 0.01
Total	24.44	34.56	13.08	2.57	12.43	7.95	13.99	0.12	5.84	1.96

Compound	K	L	M	N	O	P	Q	R	S	T

Glucoalyssin	0.55 ± 0.01	0.12 ± 0.00	0.00	1.22 ± 0.01	0.28 ± 0.00	0.45 ± 0.01	0.02 ± 0.00	0.07 ± 0.00	0.25 ± 0.01	0.03 ± 0.00
Gluconapin	1.13 ± 0.02	0.33 ± 0.01	0.00	1.07 ± 0.02	0.57 ± 0.01	0.31 ± 0.02	0.00	0.00	0.12 ± 0.00	0.00
Glucobrassicanapin	2.62 ± 0.01	0.93 ± 0.02	0.00	2.85 ± 0.01	0.99 ± 0.04	0.72 ± 0.01	0.00	0.00	8.22 ± 0.05	0.00
Glucobrassicin	0.09 ± 0.00	0.00	0.02 ± 0.00	0.01 ± 0.00	0.00	0.00	0.01 ± 0.00	0.33 ± 0.01	0.15 ± 0.00	0.01 ± 0.00
4-Methoxyglucobrassicin	5.95 ± 0.03	4.19 ± 0.01	1.32 ± 0.01	4.97 ± 0.04	3.05 ± 0.03	2.25 ± 0.05	1.50 ± 0.01	5.60 ± 0.05	1.88 ± 0.03	2.26 ± 0.01
Total	10.34	5.58	1.34	10.12	4.89	3.73	1.53	6.00	10.63	2.30

## References

[1] Agerbirk N., Olsen C. E. (2012). Glucosinolate structures in evolution. *Phytochemistry*.

[2] Ko J. A., Kim W. Y., Park H. J. (2012). Effects of microencapsulated Allyl isothiocyanate (AITC) on the extension of the shelf-life of Kimchi. *International Journal of Food Microbiology*.

[3] Vaughn S. F., Berhow M. A. (2005). Glucosinolate hydrolysis products from various plant sources: pH effects, isolation, and purification. *Industrial Crops and Products*.

[4] Choi M. M. F., Liang M. M. K., Lee A. W. M. (2005). A biosensing method with enzyme-immobilized eggshell membranes for determination of total glucosinolates in vegetables. *Enzyme and Microbial Technology*.

[5] Stančík L., Macholán L., Pluháček I., Scheller F. (1995). Biosensing of rapeseed glucosinolates using amperometric enzyme electrodes based on membrane‐bound glucose oxidase or tyrosinase. *Electroanalysis*.

[6] Keum Y.-S., Jeong W.-S., Tony Kong A. N. (2004). Chemoprevention by isothiocyanates and their underlying molecular signaling mechanisms. *Mutation Research*.

[7] Thornalley P. J. (2002). Isothiocyanates: mechanism of cancer chemopreventive action. *Anti-Cancer Drugs*.

[8] Hayes J. D., Kelleher M. O., Eggleston I. M. (2008). The cancer chemopreventive actions of phytochemicals derived from glucosinolates. *European Journal of Nutrition*.

[9] van Poppel G., Verhoeven D. T. H., Verhagen H., Goldbohm R. A. (2000). Brassica vegetables and cancer prevention: epidemiology and mechanisms. *Advances in Experimental Medicine and Biology*.

[10] London S. J., Yuan J.-M., Chung F.-L. (2000). Isothiocyanates, glutathione S-transferase M1 and T1 polymorphisms, and lung-cancer risk: a prospective study of men in Shanghai, China. *The Lancet*.

[11] Giovannucci E., Rimm E. B., Liu Y., Stampfer M. J., Willett W. C. (2003). A prospective study of cruciferous vegetables and prostate cancer. *Cancer Epidemiology Biomarkers & Prevention*.

[12] Kiddle G., Bennett R. N., Botting N. P., Davidson N. E., Robertson A. A. B., Wallsgrove R. M. (2001). High-performance liquid chromatographic separation of natural and synthetic desulphoglucosinolates and their chemical vadation by UV, NMR and chemical ionisation-MS methods. *Phytochemical Analysis*.

[13] Lee K.-C., Cheuk M.-W., Chan W. (2006). Determination of glucosinolates in traditional Chinese herbs by high-performance liquid chromatography and electrospray ionization mass spectrometry. *Analytical and Bioanalytical Chemistry*.

[14] Van Doorn H. E., Van Holst G.-J., Van Der Kruk G. C., Raaijmakers-Ruijs N. C. M. E., Postma E. (1998). Quantitative determination of the glucosinolates sinigrin and progoitrin by specific antibody ELISA assays in brussels sprouts. *Journal of Agricultural and Food Chemistry*.

[15] Bennett R. N., Mellon F. A., Kroon P. A. (2004). Screening crucifer seeds as sources of specific intact glucosinolates using ion-pair high-performance liquid chromatography negative ion electrospray mass spectrometry. *Journal of Agricultural and Food Chemistry*.

[16] Hrnčiřík K., Velíšek J., Davídek J. (1998). Comparison of HPLC and GLC methodologies for determination of glucosinolates using reference material. *Zeitschrift fur Lebensmittel -Untersuchung und -Forschung*.

[17] Matthäus B., Fiebig H.-J. (1996). Simultaneous determination of isothiocyanates, indoles, and oxazolidinethiones in myrosinase digests of rapeseeds and rapeseed meal by HPLC. *Journal of Agricultural and Food Chemistry*.

[18] Wade K. L., Garrard I. J., Fahey J. W. (2007). Improved hydrophilic interaction chromatography method for the identification and quantification of glucosinolates. *Journal of Chromatography A*.

[19] Wang J., Gu H., Yu H., Zhao Z., Sheng X., Zhang X. (2012). Genotypic variation of glucosinolates in broccoli (*Brassica oleracea* var. *italica*) florets from China. *Food Chemistry*.

[20] Cataldi T. R. I., Rubino A., Lelario F., Bufo S. A. (2007). Naturally occurring glucosinolates in plant extracts of rocket salad (*Eruca sativa* L.) identified by liquid chromatography coupled with negative ion electrospray ionization and quadrupole ion-trap mass spectrometry. *Rapid Communications in Mass Spectrometry*.

[21] Lelario F., Bianco G., Bufo S. A., Cataldi T. R. I. (2012). Establishing the occurrence of major and minor glucosinolates in Brassicaceae by LC-ESI-hybrid linear ion-trap and Fourier-transform ion cyclotron resonance mass spectrometry. *Phytochemistry*.

[22] Mellon F. A., Bennett R. N., Holst B., Williamson G. (2002). Intact glucosinolate analysis in plant extracts by programmed cone voltage electrospray LC/MS: performance and comparison with LC/MS/MS methods. *Analytical Biochemistry*.

[23] Millán S., Sampedro M. C., Gallejones P. (2009). Identification and quantification of glucosinolates in rapeseed using liquid chromatography-ion trap mass spectrometry. *Analytical and Bioanalytical Chemistry*.

[24] Mohn T., Cutting B., Ernst B., Hamburger M. (2007). Extraction and analysis of intact glucosinolates—a validated pressurized liquid extraction/liquid chromatography-mass spectrometry protocol for Isatis tinctoria, and qualitative analysis of other cruciferous plants. *Journal of Chromatography A*.

[25] Rochfort S. J., Trenerry V. C., Imsic M., Panozzo J., Jones R. (2008). Class targeted metabolomics: ESI ion trap screening methods for glucosinolates based on MSn fragmentation. *Phytochemistry*.

[26] Sasaki K., Neyazaki M., Shindo K., Ogawa T., Momose M. (2012). Quantitative profiling of glucosinolates by LC-MS analysis reveals several cultivars of cabbage and kale as promising sources of sulforaphane. *Journal of Chromatography B*.

[27] Sawada Y., Akiyama K., Sakata A. (2009). Widely targeted metabolomics based on large-scale MS/MS data for elucidating metabolite accumulation patterns in plants. *Plant and Cell Physiology*.

[28] Song L., Morrison J. J., Botting N. P., Thornalley P. J. (2005). Analysis of glucosinolates, isothiocyanates, and amine degradation products in vegetable extracts and blood plasma by LC-MS/MS. *Analytical Biochemistry*.

[29] Tian Q., Rosselot R. A., Schwartz S. J. (2005). Quantitative determination of intact glucosinolates in broccoli, broccoli sprouts, Brussels sprouts, and cauliflower by high-performance liquid chromatography-electrospray ionization-tandem mass spectrometry. *Analytical Biochemistry*.

[30] Tolrà R. P., Alonso R., Poschenrieder C., Barceló D., Barceló J. (2000). Determination of glucosinolates in rapeseed and *Thlaspi caerulescens* plants by liquid chromatography-atmospheric pressure chemical ionization mass spectrometry. *Journal of Chromatography A*.

[31] Chun J. H., Kim N. H., Seo M. S., Jin M., Park S. U., Arasu M. V. (2016). Molecular characterization of glucosinolates and carotenoid biosynthetic genes in Chinese cabbage (*Brassica rapa* L. ssp. *pekinensis*). *Saudi Journal of Biological Sciences*.

[32] Park M.-W., Kim K.-H., Kim S.-H., Park K.-Y. (1997). Inhibitory effects of Kimchi extracts on carcinogen-induced cytotoxicity and transformation in C3H/10T1/2 cells. *Preventive Nutrition and Food Science*.

[33] Lee Y. M., Kwon M. J., Kim J. K., Suh H. S., Choi J. S., Song Y. O. (2004). Isolation and identification of active principle in chinese cabbage Kimchi responsible for antioxidant effect. *Korean Journal of Food Science and Technology*.

[34] Song Y.-O. The functional properties of Kimchi for the health benefits.

[35] ISO International Organization for Standardization

[36] Kusznierewicz B., Iori R., Piekarska A., Namieśnik J., Bartoszek A. (2013). Convenient identification of desulfoglucosinolates on the basis of mass spectra obtained during liquid chromatography-diode array-electrospray ionisation mass spectrometry analysis: method verification for sprouts of different *Brassicaceae* species extracts. *Journal of Chromatography A*.

